# Female community health volunteers service utilization for childhood illness- improving quality of health services only is not enough: a cross-sectional study in mid-western region, Nepal

**DOI:** 10.1186/1472-6963-14-383

**Published:** 2014-09-11

**Authors:** Moe Miyaguchi, Junko Yasuoka, Amod Kumar Poudyal, Ram Chandra Silwal, Masamine Jimba

**Affiliations:** Department of Community and Global Health, the University of Tokyo, Tokyo, Japan; Department of Community Medicine and Public Health, Tribhuvan University, Kathmandu, Nepal; Green Tara (NGO), Kathmandu, Nepal

**Keywords:** Child health services, Health care seeking behavior, Female community health workers, Nepal

## Abstract

**Background:**

Female Community Health Volunteers (FCHVs) are considered service providers for major health problems at the community level in Nepal. However, few studies have been conducted about the roles of FCHVs from the users’ perspective. This study sought to examine the current status of FCHV service utilization and identify the determinants of caregivers’ utilization of FCHVs’ health services in the mid-western region of Nepal.

**Methods:**

This cross-sectional study targeted 446 caregivers of children under five years of age and whose children had ever fallen ill in the study village development committees (VDCs) of three districts of Nepal. Caregivers were asked about their usual health practices for childhood illness, health service utilization for childhood illness, children’s health condition, satisfaction with health services, and socio-demographic status. Descriptive statistics and multiple logistic regression were used for analysis.

**Results:**

Among 446 caregivers, 66.8% had never sought care from FCHVs for their children’s illnesses in their lifetime, and more than 50% of them were unaware of FCHVs’ services for acute respiratory infection and diarrhea. Among 316 caregivers whose child had an illness during the last seven months, 92.3% of them (n = 293) did not take their child to FCHVs. The main reasons were the lack of medicine available from them and their incompetency in providing care. Among the 446 caregivers, those who participated in a mothers’ group (n = 82) were more likely to use FCHVs’ services in their lifetime (AOR = 3.23, 95% CI =1.81-5.76).

**Conclusions:**

Caregivers can gain benefit by using FCHV’s health services, but a majority of the caregivers did not seek care from FCHVs due to its limited quality. Raising caregivers’ awareness on FCHV is equally important at community level.

**Electronic supplementary material:**

The online version of this article (doi:10.1186/1472-6963-14-383) contains supplementary material, which is available to authorized users.

## Background

Child mortality still remains high in developing countries. In 2011, 6.9 million children died in the world, and an estimated 83% of under-five deaths occurred in Sub-Saharan Africa and Southern Asia [1]. Acute respiratory infection (ARI) and acute diarrheal disease (ADD) are the major killers of children under five, and both are preventable and treatable by various existing interventions including feeding practices, oral rehydration salts (ORS) and antibiotics [1, 2].

To improve treatment, a well-trained health workforce plays a key role, but the lack of such a trained health workforce has been one of the main challenges in developing countries [3]. To tackle this problem, Community Health Workers (CHWs) have been introduced into resource-limited, rural areas [4–6]. The World Health Organization (WHO) and United Nations Children’s Fund (UNICEF) have issued a policy statement to promote pneumonia and diarrhea management by CHWs [7, 8]. Among several targeted countries, several CHW interventions were successfully carried out in hard-to-reach populations in developing countries [5, 9].

Nepal is one of the countries most well positioned to achieve Millennium Development Goal 4. Over the past two decades, the country’s under-five child mortality rate has been reduced by 65%, from 158 in 1990 to 54 in 2010, a notably low rate compared to other Asian developing countries [1, 10]. To achieve this, Nepal has implemented community-based maternal and child health programs, such as immunization and micronutrient interventions, through trained Female Community Health Volunteers (FCHVs) [9, 11].

In Nepal, nearly 50,000 FCHVs have been mobilized for prevention, diagnosis, and treatment services at the community level [12, 13]. FCHVs are locally recruited women, ready to work voluntarily and usually selected by mothers’ group. After attending an 18-day training, they offer community-based health and family planning services, including safer motherhood, newborn care, immunization, nutrition counseling, communicable and epidemic disease control, and health education [12]. FCHVs also diagnose ARI and treat children with cotrimoxazole; identify ADD and treat with ORS and zinc as part of the community-based integrated management of childhood illness (CB-IMCI) program, which has reached and provided nationwide coverage since 2009 [12–14].

Several studies have confirmed the success of FCHV interventions toward improving child health and reducing child mortality in Nepal. Trained FCHVs can offer health services of almost equivalent quality to those provided by facility-based health workers; for example, they can correctly diagnose, treat, and recognize danger signs of common childhood illnesses [15]. ARI-specific mortality was reduced with community-based treatment of childhood pneumonia provided by CHWs including FCHVs in Jumla district, a remote mountainous area of Nepal [16]. Another study showed that community-based IMCI programs provided by FCHVs increased ARI case detection from 1,290,632 in 2004 to 1,498,356 in 2006. As for diarrhea, the number of severe diarrhea episodes was significantly lower in districts with community-based IMCI conducted by FCHVs than in districts without the program [17]. From 2011 to 2012, FCHVs treated 55% of ARI and 55% of diarrheal cases [18].

Despite such reported effectiveness of FCHVs, their child health services have been limited. The 2011 Nepal Demographic and Health Survey (DHS) reported that, many children’s caregivers seek treatment at health facilities, while only 3% went to FCHVs [10]. Utilization of FCHVs’ services was higher in the Nepal Family Health Program survey, which was conducted mainly in rural areas, but still only 14% of caregivers sought care from FCHVs [19]. In rural areas, most people visit traditional healers first when they fall ill [20], especially among those of low socio-economic status [21]. For childhood illness, self-medication or traditional medicine are commonly pursued treatment options, but most research has focused on health facility utilization. Also, little research has addressed how other health services, such as FCHVs and traditional healers, are utilized.

Caregivers’ health care-seeking behavior is an important factor influencing childhood illness management. In developing countries, several studies have reported a variety of determinants of health care-seeking behavior for childhood illness- including caregiver’s education level, economic status, age, and ethnicity; distance to the health facility; child’s age; child’s nutritional status; caregiver’s recognition of illness severity; caregiver’s prior participation in health education; caregiver’s knowing a medical doctor; and health care quality issues [22–26]. In Nepal, health care-seeking behaviors for childhood illnesses are known to be associated with the number of symptoms, caregiver’s level of education, family income [27], child’s gender [28], and cost of health care [29]. CHW activities and education programs, meanwhile, have improved caregivers’ care-seeking behaviors [30]. Providing antenatal health education packages through FCHVs has also shown positive results in increasing caregivers’ health care-seeking behaviors, service utilization, and recognition of danger signs [31]. Such findings suggest that FCHV programs can be effective for improving health care-seeking behaviors for childhood illnesses.

Yet few studies have focused on current status and the determinants of caregivers’ FCHV service utilization in Nepal. Thus, the objectives of this study were 1) to examine current status of FCHV service utilization, and 2) to identify the determinants of caregivers’ utilization of FCHVs’ services for their children’s illnesses, in the mid-western region of Nepal, one of the most remote and economically depressed areas of the country.

## Methods

This cross-sectional study was conducted in June 2012, in three Village Development Committees (VDCs, the smallest political unit in Nepal) of three different districts located in the mid-western hill region of Nepal: Bijayshwari VDC (Rukum district), Kalagaun VDC (Salyan district), and Jagatipur VDC (Jajarkot district). The three VDCs and districts were purposively selected for the study sites based on the following three characteristics: 1) rural mountainous areas with limited transportation, but accessible by car or plane; 2) high numbers of childhood illness cases reported in Bijayshwari VDC (the second highest cases in the Rukum district) and Jagatipur district (the third highest cases within Jajarkot VDC) in 2011; and 3) technical support was available from the Chaurjahari mission hospital, which is located at the boundary of the three VDCs. Participants in this study were the primary caregivers of children under five years of age who had ever been ill by the time of data collection. Among those participants, we also asked recent health service utilization for the primary caregivers of children under five years of age who had been ill during the past seven months. Primary caregivers were excluded from the study if they were under the age of 18 years or had serious physical or mental disability.

Sample size was calculated based on an estimated rate of utilization of FCHVs’ services for any childhood illness. The required sample size was calculated to be 240, to attain 80% power with alpha set at 0.05 for a two-tailed test. However, to counteract the effect of missing data, data were collected from 450 households.

A two-stage random sampling method was adopted. First, three wards from nine wards in each VDC were selected randomly. After selecting these wards, a name list was made of households with children under five years based on VDC name lists. Data collectors visited every household to verify which households had children under five years. From three wards of each VDC, 150 participants were randomly selected from the list. The number of participants from each ward was adjusted proportional to the total number of households therein because the number of households varied among wards. In Jagatipur VDC, however, the total household number in the selected wards was only 155, so we included every household in the study.

### Data collection procedures

In total, 455 caregivers were selected for the survey. Among them, 451 caregivers agreed to participate in the survey, but five caregivers were excluded as their children had never been ill in their lifetime, resulting in 446 participants. Out of 446 participants, 316 had children who had fallen ill within the last 7 months. These 316 participants were analyzed for health service utilization, while a total of 446 participants were analyzed for FCHV service utilization.

Primary caregivers were interviewed for approximately 40 minutes using a structured questionnaire administered by trained interviewers in the Nepali language. To this end, VDC secretaries or FCHVs were asked to invite the primary caregivers of children under five years of age to public places such as schools, where interviewers then conducted face-to-face interviews.

If a primary caregiver had more than one child under five years of age, the caregiver was asked specifically about the child who had experienced an illness most recently. If no child had fallen ill within the seven months, the youngest child was designated as the focus of the interview questions.

### Measurements

The questionnaire [Additional file 1] was adapted from the Nepal DHS questionnaire [10], IMCI Household Survey questionnaire [32], and Nepal Family Health Program survey questionnaires [19, 33]. Items elicited information on health service utilization for childhood illness including utilization of FCHVs’ services, knowledge of danger signs of childhood illness, usual health practices for childhood illness and socio-demographic characteristics.

#### Utilization of FCHVs’ services

Caregivers were asked if they had visited an FCHV for treatment of their child’s illness, especially for ARI or diarrhea [10]. If caregivers’ answer were yes, they were categorized as “FCHVs’ service user”. In addition, caregivers were asked if they were aware of their ward’s FCHVs’ other services. FCHVs’ services in our survey included providing health information through mothers’ groups, advice to pregnant women, advice to post-partum mothers, advice regarding newborn care, condom and pill supplies, vitamin A for mother and child, and information on human immunodeficiency virus/acquired immune deficiency syndrome (HIV/AIDS) and other sexually transmitted infections (STIs) [19, 33].

#### Usual health practices for childhood illness

Caregivers were asked about their usual health practices for their children’s illnesses, including usual care seeking behavior, knowledge of danger signs, distance to health services, and cost of health services. Regarding the health costs, caregivers were asked if they could usually afford the costs of health care. For the distances, they were asked how long it takes to get from their house to each health service provider [32].

#### Health service utilization for childhood illness

Caregivers were asked regarding their actual practices in seeking health services in response to their children’s illnesses. First, they were asked if their children fell ill after Dashain, one of the biggest festivals in Nepal, which took place seven months prior to the time of survey. If the answer was yes, they were also asked from where and whom they had sought treatment [32].

#### Socio-demographic characteristics

Socio-demographic characteristics were assessed using items from the DHS Household, Women’s, and Men’s Questionnaires, which include items on age, sex, education, ethnicity, religion, and family structure elements such as child’s age and gender [10]. For ethnicity, participants were classified into three broad categories: upper caste (Brahmin/Chhetri/Jogi), indigenous ethnic groups (Janajati), and lower caste (Dalit).

Economic status of households was assessed by a weighted wealth index adopted from the 2011 Nepal DHS. The index incorporated information on roofing materials, ownership of agricultural land, livestock ownership, and ownership of household assets including televisions, radios, clocks, fans, mobile phones, and dhiki (traditional wooden thresher) [8]. The variables were dichotomized and principal component analysis used as item weights that were summed to generate a wealth index. The total weighted wealth index score was subsequently divided into three categories: 40% “poor”, 40% “middle”, and 20% “rich” [34].

### Data analysis

We compared socio-demographic characteristics along with knowledge-, health-, and FCHV-related variables between caregivers who had ever used FCHVs’ services and those who never used FCHVs’ services. Health service providers were categorized into three groups: health facilities, pharmacies, or FCHVs. Health facilities encompassed private hospitals, public hospitals, and health posts (or sub-health posts).

Chi-square or Fisher’s exact test were applied to test the significant differences as appropriate. In addition, multiple logistic regression analysis was conducted to examine determinants of utilization of FCHVs’ services for childhood illness. We controlled for economic status, number of family members, living with grandparents, caregivers’ age, literate ability, caste, able to pay the cost of health care, autonomy for health service utilization, past experience for mothers’ group participation, time to FCHV’s residence, time to any health facilities, and satisfaction with FCHVs’ services. Multicolliniearity was also checked by examining Spearman’s correlation coefficient, and groups of correlated variables were defined using an absolute rho value = 0.5 or more.

For all procedures, statistical significance was set at p-value less than 0.05. Statistical analysis was performed using the Stata Special Edition 11.2 software package (StataCorp, College Station, Texas, USA).

### Ethical considerations

Ethical approval was obtained from the Nepal Health Research Council (NHRC) and the Research Ethics Committee of the Graduate School of Medicine, the University of Tokyo. Primary caregivers participated voluntarily, and the confidentiality of their answers was maintained throughout the survey. Before administering the interview, informed consent was obtained in written form from all participants and with thumbprints from those who were illiterate.

## Results

### Differences in characteristics between FCHVs’ services users and non-users

Out of 446 participants, almost all primary caregivers were mothers (94.2%), married (98.0%), and believed in the Hindu religion (97.3%). The mean numbers of their family members and children were 5.5 (standard deviation [SD] 1.8, 95% CI = 3-9) and 2.5 (SD 1.3, 95% CI = 1-7), respectively. Mean age of participants was 26.9 (SD 8.4, 95% CI = 18-65) years old; 61% had no formal schooling (N = 268) and 34.3% were illiterate (N = 153; Table 1). Among participants, 66.8% had never utilized FCHVs’ services for their children’s illnesses in their lifetime.

**Table 1:** **Socio-demographic characteristics of respondents by FCHVs’ services utilization (N = 446)**

	FCHVs’ service user		Non-user		
Variable	(n = 446)	%	(n = 298)	%	p-value
Number of family members^†^					
3 or 4	46	31.1	92	30.9	0.964
5 or more	102	68.9	206	69.1	
Number of children^†^					
1	39	26.4	85	28.5	0.890
2	39	26.4	76	25.5	
3	70	47.3	137	46.0	
Living with grandparents^†^					
Yes	47	31.8	123	41.3	0.051
Caregiver’s age^†^					
< 25	143	48.0	68	46.0	0.684
25 or more	155	52.0	80	54.0	
School education level (no. of years)^†^					
Never attended	81	54.7	187	62.8	0.253
1-5	17	11.5	26	8.7	
6 or more	50	33.8	85	28.5	
Literacy^†^					
Literate	101	68.2	192	64.4	0.424
Illiterate	47	31.8	106	35.6	
Caste‡					
Upper caste	93	62.8	205	68.8	0.451
Janajati (indegious)	11	7.4	18	6.0	
Dalit	44	29.7	75	25.2	
Economic status^†^					
Poor	58	39.2	120	40.3	0.969
Middle	60	40.5	120	40.3	
Rich	30	20.3	58	19.5	
Decision maker about child health care					
Mother can decide^†^	125	84.5	279	93.6	0.002**
Mother-in-law decides^†^	28	18.9	45	15.1	0.515
Able to pay costs for health care^†^					
Yes/Usually	109	73.7	276	92.6	<0.001**
Have health facility within 1 hour walk^†^					
Yes	100	67.6	167	56.0	0.019*
Time to FCHV's house^†^					
Less than 10 minutes	77	52.0	86	28.9	<0.001**
11-30 minutes	49	33.1	118	39.6	
More than 30 minutes	22	14.9	94	31.5	
Have participated in mothers' group^†^	50	33.8	32	10.7	<0.001**

^†^, Chi-square test; ^‡^, Fisher’s exact test.

*, p < 0.05; **, p < 0.01.

No significant difference was detected in socio-demographic characteristics between caregivers who had ever used FCHVs’ services and those who had never used such services. However, FCHVs’ services users’ autonomy for decision making and affordability were significantly lower compared to non-users’. A significantly lower percentage of FCHVs’ services users could decide to go to a health facility of their own independent accord (84.5% vs. 93.6%. p = 0.002). Also, they were less frequently able to cover health care costs compared to non-users (73.7% among FCHVs’ services users vs. 92.6% among non-users, p < 0.001).

FCHVs’ services users tended to spend less time to reach health services compared with non-users. Also relative to non-users, a significantly higher percentage of FCHVs’ services users had used a private hospital (42.6% vs. 24.2%, p < 0.001), health post (29.7% vs. 21.5%, p < 0.001), or pharmacy (64.2% vs. 50.7%, p = 0.026) within one hour of their residences. Moreover, a higher percentage of FCHVs’ services users lived within a 10-minute walk of an FCHV’s residence compared to non-users (52.0% vs. 28.9%, p < 0.001).

About half of caregivers were not aware of FCHVs’ services, and about one-third of caregivers received services from FCHVs (Table 2). The vitamin A program is an exception, with 90% of both users and non-users receiving vitamin A from FCHVs. FCHVs’ services users exhibited a significantly higher percentage of FCHVs’ services awareness, service utilization, and FCHVs’ services satisfaction. Significantly higher percentage of FCHVs’ services users were extremely satisfied with FCHVs’ services compared to non-users (33.1% vs. 20.1%, p < 0.001).

**Table 2:** **Respondents' knowledge and utilization of FCHVs’ services by FCHVs’ services utilization (N = 446)**

	FCHVs’ services user		Non-user		
Variable	(n = 148)	%	(n = 298)	%	p-value
Knowledge about danger signs					
Fever^†^	135	91.2	281	94.3	0.222
Respiratory symptom^†^	116	78.4	202	67.8	0.020*
Diarrheal symptom^†^	81	54.7	125	41.9	0.011*
Knowledge about FCHV-provided services					
Vitamin A dispensing^†^	139	93.9	273	91.6	0.387
Iron tablet dispensing^†^	94	63.5	137	46.0	<0.001**
Advice for pregnant woman^†^	100	67.6	125	42.0	<0.001**
Advice for post-partum woman^†^	90	60.8	120	40.3	<0.001**
Advice regarding newborn care^†^	82	55.4	107	35.9	<0.001**
Treatment for diarrhea^†^	99	66.9	103	34.6	<0.001**
Condom and pill dispensing^†^	75	50.7	124	41.6	0.070
Treatment for ARI^†^	58	39.2	92	30.8	0.080
Health information^†^	78	52.7	109	36.6	0.001**
HIV/AIDS/STI information^†^	32	21.6	58	19.5	0.593
Services received from FCHV					
Vitamin A dispensing^†^	134	90.5	270	90.6	0.983
Iron tablet dispensing^†^	73	49.3	96	32.2	<0.001**
Advice for pregnant woman^†^	59	39.9	13	4.4	<0.001**
Advice for post-partum woman^†^	48	32.4	8	2.7	<0.001**
Advice regarding newborn care^†^	53	35.8	9	3.0	<0.001**
Condom and pill dispensing^†^	46	31.1	15	5.0	<0.001**
Health information^†^	34	23.0	6	2.0	<0.001**
HIV/AIDS/STI information^†^	23	15.5	6	2.0	<0.001**
Satisfaction with FCHVs’ services^‡^					
Extremely satisfied	49	33.1	60	20.1	<0.001**
Generally satisfied	95	64.2	178	59.7	
Not satisfied	4	2.7	49	16.4	
Never met FCHV	0	0.0	11	3.7	
Things that need to be improved about FCHVs’ services					
Medicine availability^†^	99	66.9	182	61.1	0.472
Service quality^†^	50	33.8	87	29.2	0.460
Interpersonal manners^†^	23	15.5	79	26.5	0.005**
Access^†^	33	22.3	65	21.8	0.934
Health advice^†^	7	4.7	12	4.0	0.807

^†^, Chi-square test; ^‡^, Fisher’s exact test.

*, p < 0.05; **, p < 0.01.

### Factors associated with utilization of FCHVs’ services for childhood illness

Multiple logistic regression analysis was used to analyze factors associated with utilization of FCHVs’ services (Table 3). Children who lived with grandparents were 52% less likely to have consulted FCHVs (95% CI = 0.27-0.86). Likewise, participants who were able to cover health care costs and who had autonomy in child health matters were 76% (95% CI = 0.12-0.46) and 58% (95% CI = 0.20-0.89) less likely, respectively, to have used the services of FCHVs. Furthermore, time taken to reach FCHV’s residence was also negatively associated with FCHVs’ services utilization. Participants who could reach FCHV’s residence by walking within 11-30 minutes were 58% less likely (95% CI = 0.25-0.70) to have ever used FCHVs’ services, and those who had to walk more than 30 minutes to reach FCHV’s residence were 69% less likely (95% CI = 0.16-0.59) to have ever used FCHVs’ services than those who could reach FCHV’s residence within just 10 minutes. On the other hand, participants who had ever participated in a mothers’ group were 3.2 times more likely to have used FCHVs’ services (95% CI = 1.81-5.76).

**Table 3:** **Association between selected socio-demographic factors and FCHVs’ services utilization (N = 446)**

			95% CI	
Variable		AOR	Lower	Upper
Economic status	Poor			
	Middle	1.19	0.68	2.06
	Rich	1.52	0.76	3.04
Number of family members	3 or 4			
	5 or more	1.38	0.77	2.47
Living with grandparents		0.48*	**0.27**	**0.86**
Caregiver’s age	25 or more			
	Less than 25	1.14	0.69	1.90
Literate		1.04	0.81	1.33
Caste	Upper caste			
	Dalit	1.27	0.73	2.19
	Janajati (indigenous)	1.19	0.48	2.91
Able to pay the cost of health care				
	Always/usually	0.24**	**0.12**	**0.46**
Mother can decide to use health service				
	Yes	0.42*	**0.20**	**0.89**
Have participated in mothers’ group				
	Yes	3.23**	**1.81**	**5.76**
Time to FCHV's residence	Less than 10 minutes			
	11-30 minutes	0.42*	**0.25**	**0.70**
	More than 30 minutes	0.31**	**0.16**	**0.59**
Have health facility within one hour’s walk				
	Yes	1.63	0.99	2.68
Satisfaction with FCHVs’ services	Generally satisfied/not satisfied			
	Extremely satisfied	1.55	0.93	2.57

*, p < 0.05; **, p < 0.01.

### Caregivers’ recent care seeking behaviors for childhood illness

Among 446 children, 316 experienced any illness during the past seven months. Their reported common symptoms were fever (82.3%), respiratory symptoms (73.7%), and diarrheal symptoms (41.8%). About one-third (32.3%) of caregivers recognized more than three of these symptoms, and 52.5% recognized their children’s illnesses as severe.

Of those 316 children who had fallen ill in the last seven months, seven children did not visit any health service provider for treatment. Of the remaining 309 children who did seek treatment, 36 (11.7%) went to a health facility, 249 (80.6%) visited a pharmacy, and 23 (7.4%) consulted a FCHV (Table 4). Seventy-four caregivers (23.4%) took their child to more than one health service provider, and 13 (4.1%) of them went to three providers. FCHVs, where consulted, were always consulted as the first-choice health service provider, and all FCHVs’ services users also visited at least one other provider. Among caregivers who did not visit FCHVs for their children’s illness during the last seven months (n = 293), two major reasons for not using FCHVs’ services were “FCHVs often did not have medicine” (55.4%) and “FCHVs were not competent” (26.3%).

**Table 4:** **Health service utilization and satisfaction among caregivers of children who had fallen ill during the last 7 months (n =C316)**

	Health facility		Pharmacy		FCHV		p-value
Variable	(n = 36)	%	(n = 249)	%	(n = 23)	%	
Satisfaction with first-choice health service provider^†^							
Extremely satisfied	13	36.1	109	43.8	2	8.7	0.008**
Generally satisfied	21	58.3	128	51.4	19	82.6	
Not satisfied	2	5.6	12	4.8	2	8.7	
Visited second-choice health service provider^†^							
Yes	19	54.8	31	14.2	23	100.0	<0.001**
Reason for not visiting FCHV							
FCHV had no medicine^†^	18	50.0	155	62.3			0.160
FCHV was not competent^†^	17	47.2	64	25.7			0.007**
Distance^†^	7	19.4	41	16.5			0.091
Did not know FCHV providing treatment^‡^	1	2.8	6	2.4			1.000
Benefit of getting treatment from FCHVs							
Saves time^†^	14	38.9	73	29.3	10	43.5	0.224
Able to acquire medicine^‡^	3	8.3	24	10.7	7	30.4	0.015*
Free of cost^‡^	1	2.8	13	5.2	2	8.7	0.620

^†^, Chi-square test; ^‡^, Fisher’s exact test.

*, p < 0.05; **, p < 0.01.

## Discussion

This study showed that caregivers underutilized health services of FCHVs when their children suffered from illness in the study region. Major factors for the low utilization were lack of medicine with FCHV, perceived incompetency of FCHVs to provide services, and lack of awareness about FCHVs’ services. The study also suggested that FCHVs’ services were underutilized for other services in pregnancy, delivery, and postpartum and newborn care. However, caregivers’ participation in mothers’ groups was positively associated with their utilization of FCHVs’ services.

Most caregivers did not visit FCHVs for their children’s illness in the study site. Only 33% of caregivers had ever utilized FCHVs’ services in their lifetime, and less than 10% of caregivers utilized FCHVs’ services when their children had fallen ill during the past seven months. Underutilization of FCHVs’ child health program was consistent with previous DHS data [8], but caregivers’ awareness about FCHVs’ services was much lower in this study than indicated in the previous national study [19]. Possible reasons for underutilization of FCHVs’ services include caregivers’ lack of awareness about FCHVs’ services, lack of medicine with FCHVs, and easy access to pharmacies, from which caregivers could obtain a variety of medicines and advice.

Medicine availability was the main concern for caregivers. More than 50% of caregivers answered lack of medicine was the reason not to visit FCHVs. Also, about 65% of caregivers pointed out medicine availability should be improved in FCHVs’ services. Although medicines were supplied to FCHVs through health posts, the supply was frequently delayed, which often led to shortages of medicines and other commodities at FCHVs [35–37]. If the district and VDC could procure and supply drugs promptly, FCHVs’ services might be more widely utilized by caregivers in Nepal.

Many caregivers also claimed that FCHVs skill, knowledge, and attitude were not friendly to the client. Notably, 31.5% and 23.5% of caregivers raised FCHVs’ inefficient service quality and FCHVs’ lack of interpersonal manners as their primary concern, respectively. These were also claimed in the past as reasons for not using FCHVs’ services [19]. The low satisfaction with FCHVs’ services might have reflected caregivers’ evaluations of FCHVs’ insufficient capability. Providing additional and ongoing training for FCHVs might help to improve their competence, increase satisfaction with their health services among caregivers, and thus increase the utilization of their services. Previous research conducted in the eastern region of Nepal reported that FCHVs were able to acquire more knowledge and skills on several neonatal health services by attending additional training [16]. Another study conducted in the same region reported that caregivers were more likely to have visited FCHVs for child health consultations after attending education programs provided by FCHVs and other health personnel [38]. Such findings suggest that providing better and further training for FCHVs would improve their skills along with caregivers’ care seeking behaviors.

Only the vitamin A program was well recognized among caregivers as one of the health services offered by FCHVs. While only about a third of caregivers had ever used FCHVs’ services for their children’s illnesses, 90% of caregivers had used the vitamin A program. The Nepal National Vitamin A Program (NVAP) was expanded nationwide in 2002 and is now widely recognized among caregivers because of the government’s promotion efforts. This program can serve as a model for CB-IMCI program to increase awareness about their initiatives and promote utilization of FCHVs among caregivers. In the NVAP program, vitamin A capsules are provided in every six months to children aged 6-59 months through FCHVs [31, 35, 36]. NVAP uses mass media to promote the program and to have FCHVs deliver information to residents in their communities regarding time and location of the vitamin A distribution campaigns [37]. Such an active promotion of CB-IMCI programs could similarly encourage caregivers to utilize FCHVs’ services for childhood illness management.

Caregivers’ participation in mothers’ groups had a strong impact on FCHVs’ services utilization. Mothers’ groups are comprised of local women of reproductive age and promote cultural, income-generating, and health promotion programs within their own communities. As members of the mothers’ group, FCHVs play an important role in motivating and educating local mothers and community members. Through a mothers’ group, caregivers will get information about health issue and available health services from FCHVs. However, mothers’ group participation rates were not high in the study site - only 18.4% of all caregivers had ever participated. Encouraging caregivers to participate in a mothers’ group may be effective to increase utilization of FCHVs’ services and caregivers’ awareness of FCHVs’ services.

In Nepal, FCHVs’ service quality was not at a sufficient level in many communities [36, 38]. Additional training should be provided to FCHVs, to improve the quality of their services and the maternal and child health status of the region. This is because knowledgeable FCHVs can attract the larger numbers of caregivers who consult FCHVs for ARI [39]. FCHVs’ services might be effectively expanded through training. To strengthen communities’ health services, it would be ideal to train FCHVs to promote and provide CB-IMCI services more effectively.

The study results need to be interpreted in light of certain limitations. First, reporting and recall biases might have occurred because we relied on respondents’ self-report about their children’s illnesses during the past seven months. However, attempts were made to minimize these potential biases by pre-testing the questionnaire and training interviewers. Also, we used the Nepali calendar and set the reference point for retrospective recollection to fall just after the major Nepali festival of Dashain in order to stimulate participants’ memories. Second, it may be difficult to generalize the study results nationwide because the study districts were chosen purposively.

Despite these limitations, this is one of the few studies to describe how health services are being utilized for childhood illness, including determinants for utilization of FCHVs’ services in the mid-western region of Nepal. In addition, to the best of our knowledge, no other studies have highlighted underutilization of FCHVs’ services in rural areas after the CB-IMCI program’s introduction, though FCHVs have since spread throughout the country.

## Conclusions

Caregivers underutilizes FCHVs’ treatment services of childhood illnesses in rural Nepal. The main reasons for such underutilization are found both among service providers and users; 1) medicines were not available through FCHVs’ services, 2) FCHVs were not competent, and 3) caregivers are not aware of FCHVs’ services. Appropriate training is needed for FCHVs but awareness raising is also necessary for caregivers to improve health services. This should be accompanied by training service providers in interpersonal relations and an assurance of regular supply of medicines to FCHVs so that when caregivers decide to seek care the service is readily available.

## Electronic supplementary material

Additional file 1: **Survey questionnaire.** (DOCX 237 KB)

## Abbreviations

ADD: Acute Diarrheal Disease
ARI: Acute Respiratory Infection
CB-IMCI: Community-based Integrated Management of Childhood Illness
CHW: Community Health Workers
DHS: Demographic and Health Survey
FCHV: Female Community Health Volunteer
NVAP: Nepal Vitamin A Program
ORS: Oral Rehydration Salts
UNICEF: The United Nations Children’s Fund
VDC: Village Development Committee
WHO: World Health Organization.
