# Supplementary material for: Female community health volunteers service utilization for childhood illness- improving quality of health services only is not enough: a cross-sectional study in mid-western region, Nepal
Source: BMC Health Serv Res. 2014 Sep 11;14:383. doi: 10.1186/1472-6963-14-383 (PMC4168066; doi:10.1186/1472-6963-14-383)
Supplement: Supplementary file 1 — Additional file 1: Survey questionnaire. (DOCX 237 KB) [file 12913_2013_3480_MOESM1_ESM.docx]

**Questionnaire**

INTRODUCTION AND CONCENT

Namaste! My name is . We are from the University of Tokyo in Japan. We are here to find out about the health service utilization for childhood illnesses, particularly of children less than five years old. We would appreciate your participation in this survey. Your information will help us improve health service in your region. This interview will take about one hour.

Before starting interview, I want you to understand about this study and get your agreement for participation.

May I explain about this research? Do you agree to participate in this research?

(If respondent agrees, please show the information sheet to her/him. (if she/he does not read, please read it for her/him). Then, get signature or thumb print on the Informed consent. After getting informed consent, proceed to questionnaire)

| IDENTIFICATION | | | |
| --- | --- | --- | --- |
| Name of VDC |  | | |
| Ward number |  | | |
| Village name |  | | |
| ID of the respondent |  | | |
| Interview status | | | |
| Date | |  | DD  MM  YYYY |
| Interviewer’s name | |  |  |
| Record the time. | **Start interview**  HOUR 　　　　　　　　　 MINUTES    **Finish Interview**  HOUR 　　　　　　　　　 MINUTES | | |

| **Household Roster** | | | | | | | | | | | | | | | | | | | | | | | | | | | | | | | | | | | | | | | | | | | | | | | | | | | | | | | |
| --- | --- | --- | --- | --- | --- | --- | --- | --- | --- | --- | --- | --- | --- | --- | --- | --- | --- | --- | --- | --- | --- | --- | --- | --- | --- | --- | --- | --- | --- | --- | --- | --- | --- | --- | --- | --- | --- | --- | --- | --- | --- | --- | --- | --- | --- | --- | --- | --- | --- | --- | --- | --- | --- | --- | --- |
| Please tell me the name and sex of each person who lives here, starting with the head of the household. | | | | | | | | | | | | | | | | | | | | | | | | | | | | | | | | | | | | | | | | | | | | | | | | | | | | | | | |
| No. | | Usual residents | Relationship to head of household | | | sex | age | | | | | | | Marital status  (if age 15 or older) | | | | | | | | | | | | | | | | | Eligibility | | | | | | | | | | | | | | | | | | | | | | | | |
|  | | Please give me the IDs of the persons who usually live in your household starting with the head of the household. | What is the relationship of (name) to the head of the household? | | | Is (name) male or female? | How old is (name)? | | | | | | | What is (name’s) current marital status?  1= Currently married  2= married, but gauna not performed  3= Divorced/ Separated  4= Widowed  5=Never Married  6= Don’t know | | | | | | | | | | | | | | | | | | Circle　line number of all children under 5. | | | | | | | | | | | | | | | | Circle line number of the respondent. | | | | | | | |
| (1) | | (2) | (3) | | | (4) | (5) | | | | | | | (6) | | | | | | | | | | | | | | | | | | (7) | | | | | | | | | | | | | | | | (8) | | | | | | | |
| 01 | |  |  | | | M F  1 2 | In Years | | | | | | |  | | | | | | | | | | | | | | | | | | 01 | | | | | | | | | | | | | | | | 02 | | | | | | | |
| 02 | |  |  | | | M F  1 2 | In Years | | | | | | |  | | | | | | | | | | | | | | | | | | 01 | | | | | | | | | | | | | | | | 02 | | | | | | | |
| 03 | |  |  | | | M F  1 2 | In Years | | | | | | |  | | | | | | | | | | | | | | | | | | 01 | | | | | | | | | | | | | | | | 02 | | | | | | | |
| 04 | |  |  | | | M F  1 2 | In Years | | | | | | |  | | | | | | | | | | | | | | | | | | 01 | | | | | | | | | | | | | | | | 02 | | | | | | | |
| 05 | |  |  | | | M F  1 2 | In Years | | | | | | |  | | | | | | | | | | | | | | | | | | 01 | | | | | | | | | | | | | | | | 02 | | | | | | | |
| 06 | |  |  | | | M F  1 2 | In Years | | | | | | |  | | | | | | | | | | | | | | | | | | 01 | | | | | | | | | | | | | | | | 02 | | | | | | | |
| 07 | |  |  | | | M F  1 2 | In Years | | | | | | |  | | | | | | | | | | | | | | | | | | 01 | | | | | | | | | | | | | | | | 02 | | | | | | | |
| 08 | |  |  | | | M F  1 2 | In Years | | | | | | |  | | | | | | | | | | | | | | | | | | 01 | | | | | | | | | | | | | | | | 02 | | | | | | | |
| 09 | |  |  | | | M F  1 2 | In Years | | | | | | |  | | | | | | | | | | | | | | | | | | 01 | | | | | | | | | | | | | | | | 02 | | | | | | | |
| 10 | |  |  | | | M F  1 2 | In Years | | | | | | |  | | | | | | | | | | | | | | | | | | 01 | | | | | | | | | | | | | | | | 02 | | | | | | | |
|  | |  |  | | |  |  | | | | | | |  | | | | | | | | | | | | | | | | | |  | | | | | | | | | | | | | | | |  | | | | | | | |
|  | |  |  | | |  |  | | | | | | |  | | | | | | | | | | | | | | | | | |  | | | | | | | | | | | | | | | |  | | | | | | | |
|  | |  |  | | |  |  | | | | | | |  | | | | | | | | | | | | | | | | | |  | | | | | | | | | | | | | | | |  | | | | | | | |
| **SECTION 1 :Household Characteristics** | | | | | | | | | | | | | | | | | | | | | | | | | | | | | | | | | | | | | | | | | | | | | | | | | | | | | | | |
| **Question and Filters** | | | | | | | | | | | | | | | | | **Coding categories** | | | | | | | | | | | | | | | | | | | | | | | | | | | | | | | | | | | **Skip** | | | |
| 1 | Which items from following does your household have?  Electricity?  A radio?  A mobile phone?  A landline telephone?  A table?  A chair?  A bed?  A cupboard?  A watch/clock?  A fan?  A dhiki/Janto? | | | | | | | | | | | | | | | | Electricity  A radio  A mobile phone  A landline telephone  A table  A chair  A bed  A cupboard  A watch/clock  A fan  A dhiki/Janto | | | | | | | | | | | | Yes  1  1  1  1  1  1  1  1  1  1  1 | | | | | | | | | | | | No  2  2  2  2  2  2  2  2  2  2  2 | | | | | | | | | | |  | | | |
| 2 | What is your house’s roof mainly made of? | | | | | | | | | | | | | | | | Thatched/palm leaf ……………..  Stone …………………………….  Mud ………………………………  Galvanized sheet ……………….  Cement …………………………..  Ceramic tiles …………………….  Other(Specify) | | | | | | | | | | | | | | | | | | | | | | | | 1  2  3  4  5  6  9 | | | | | | | | | | |  | | | |
| 3 | Does any member of the family own any agricultural land? | | | | | | | | | | | | | | | Yes………………………………..  No…………………..…………….. | | | | | | | | | | | | | | | | | | | | | | | | | 1  2 | | | | | | | | | | | 5 | | | |
| 4 | How many months will the production be sufficient? | | | | | | | | | | | | | | | Less than 3 months ……………..  3-6 months ……………………....  6-12 months ……………………..  More than 12 months …………..  Don’t know ………………………. | | | | | | | | | | | | | | | | | | | | | | | | | 1  2  3  4  9 | | | | | | | | | | |  | | | |
| 5 | Does this household own any livestock, herds , other farm animals, or poultry? | | | | | | | | | | | | | | | Yes………………….……………..  No…………………...................... | | | | | | | | | | | | | | | | | | | | | | | | | 1  2 | | | | | | | | | | | 7 | | | |
| 6 | What kind of animal and how many animals does your household own? If yes, please specify how many.  If None enter ‘00’ If Unknown , enter ‘98’  Buffalo  Milk cows or bulls  Horses, donkeys, or mules  Goats  Sheep  Chickens  Pigs  Others(Specify) | | | | | | | | | | | | | | | Buffalo…………………………….  Milk cows or bulls………………..  Horses, donkeys, or mules……..  Goates……………………………  Sheep…………………………….  Chickens………………………….  Pigs……………………………….  Others (specify) | | | | | | | | | | | | | | | | | | | | | | | | |  | | | | | | | | | | |  | | | |
| SECTION 2: Respondent’s background | | | | | | | | | | | | | | | | | | | | | | | | | | | | | | | | | | | | | | | | | | | | | | | | | | | | | | | |
| 7 | | Have you ever attended school? | | | | | | | | | | | | | | | | | Yes ………………………………  No ……...……………………….. | | | | | | | | | | | | | | | | | | | | | | | | | 1  2 | | | | | | | | | 9 | | |
| 8 | | What is the highest grade you completed? | | | | | | | | | | | | | | | | | Grade ………………..………… | | | | | | | | | | | | | | | | | | | | | | | | |  | | | | | | | | | 10 | | |
|  |  | Grade 5 or lower 　　　 Grade 6 or higher | | | | | | | | | | | | | | | | | | | | | | | | | | | | | | | | | | | | | | | | | | | | | | | | | | |  | | |
| 9 | | Now I would like you to read this sentence to me.  SHOW CARD TO RESPONDENT.  IF RESPONDENT CANNOT READ WHOLE SENTENCE, PROBE:  Can you read any part of the sentence to me? | | | | | | | | | | | | | | | | | Cannot read at all ……………...  Able to read only parts of  sentence………………………...  Able to read whole sentence …  Blind/ visually impared………… | | | | | | | | | | | | | | | | | | | | | | | | | 1  2  3  9 | | | | | | | | |  | | |
| 10 | | What is your religion? | | | | | | | | | | | | | | | | | Hindu ……………………………  Christian ………………………..  Other (specify); | | | | | | | | | | | | | | | | | | | | | | | | | 1  2  9 | | | | | | | | |  | | |
| 11 | | What is your caste/ ethnicity? | | | | | | | | | | | | | | | | | Brahmin…………………………  Chhetri ……………….…………  Dalit ……………………………..  Jogi ……………………………...  Other (specify): | | | | | | | | | | | | | | | | | | | | | | | | | 1  2  3  4  9 | | | | | | | | |  | | |
| 12 | | Which of the following things can you decide by yourselves?  How your (husband’s/ partner’s) earnings will be used?  Making major household purchases?  Visit to your family or relatives?  About health care for yourself?  About health care for your children? | | | | | | | | | | | | | | | | | Earning  Purchases Visit  Your health  Child health | | | | | | | Can decide  1  1  1  1  1 | | | | | | | | | | Can not  decide  2  2  2  2  2 | | | | | | | | | | | | | | | | |  | | |
| SECTION 3: Child illness information | | | | | | | | | | | | | | | | | | | | | | | | | | | | | | | | | | | | | | | | | | | | | | | | | | | | | | | |
| 13 | | Is there someone in the community who is regularly consulted with regard to childhood illness? | | | | | | | | | Yes ……………………………..……………….  No …………………………..…………………  Don’t know …………………………………….. | | | | | | | | | | | | | | | | | | | | | | | | | | | | | | | | | 1  2  9 | | | | | | | | | | | 15 |
| 14 | | Who is this person?  DO NOT PROMPT. | | | | | | | | | Spouse …………………………………………  Community Health Worker …………………...  Relative ………………………………………...  Traditional Birth Attendant ……………………  Neighbour ………………………….…………..  Traditional Healer…………………..………….  Elder ……………………………………………  Religious Leader ………………………………  Medical Doctor ………………………………...  AMN/CMN/Nurse ……………………………...  Other (specify): …… | | | | | | | | | | | | | | | | | | | | | | | | | | | | | | | | | A  B  C  D  E  F  G  H  I  J  X | | | | | | | | | | |  |
| 15 | | When should you take a child to a health facility right away? | | | | | | | | | Child not able to drink or breastfeed ………….  Child becomes sicker …………………………..  Child develops a fever …………………………  Child has fast breathing ………………………..  Child has blood in stool ………………………...  Child is drinking poorly …………………………  Child vomiting everything ……………………...  Child has Diarrhoea …………………………….  Child has ARI ………………….........................  Other (Specify) …………..  Never take the child to a health facility ………. | | | | | | | | | | | | | | | | | | | | | | | | | | | | | | | | | | | A  B  C  D  E  F  G  H  I  X  Y | | | | | | | | |  |
| 16 | | If your child have illness (fever, ARI, diarrhea), do you seek treatment ourside ? | | | | | | | | | Fever ……………….  ARI ………………….  Diarrhea …………… | | | | | | | | | | | | Yes  1  1  1 | | | | | | | | | | | No  2  2  2 | | | | | | | | | | | | | | | If circle No for every illness, go to Q19 | | | | | | |
| 17 | | If Yes;  Where do you usually seek treatment for fever/ARI/diarrhea? | | | | | | | | | Mission Hospital …….  Health Post/  Sub health Post ……..  PHC Outreach Clinic..  FCHV ………………...  Pharmacy ……………  Ayurbedic clinic………  Traditional healer ……  Other(specify) | | | | | | | | | | | | | Fever  A  B  C  D  E  F  G  H | | | | | | | | | ARI  A  B  C  D  E  F  G  H | | | | | | | | | | Diarrhea  A  B  C  D  E  F  G  H | | | | | | | | | | | |  |
|  |  | FCHV not circled FCHV circled | | | | | | | | | | | | | | | | | | | | | | | | | | | | | | | | | | | | | | | | | | | | | | | | | | | | 19 | |
| 18 | | Have you ever seek treatment from FCHVs for any illness? | | | | | | | | | | | Yes ………………………………………….  No ……………………………………………  Don’t know …………………………………. | | | | | | | | | | | | | | | | | | | | | | | | | | 1  2  9 | | | | | | | | | | | | | | |  | |
| 19 | | After Dashain, has any child had any illness or health problems?  If yes, please write the age of that child: | | | | | | | | | YES ………………………….……………….  NO ………………………………………….  DON’T KNOW ………………...................... | | | | | | | | | | | | | | | | | | | | | | | | | | | | 1  2  9 | | | | | | | | | | | | | | | 47 | |
| 20 | | If Yes, when and how many days has your child been ill?  (SHOW CALENDER TO RESPONDENT)  IF CHILD GOT ILLNESS MORE THAN TWO TIMES, ASK ABOUT MOST RECENT ILLNESS. | | | | | | | | | Date .............  Don’t remember the date …………………..  No. of Days:  Don’t remember how many days ……….. | | | | | | | | | | | | | | | | | | | | | | | | | | | | 9  99 | | | | | | | | | | | | | | |  | |
| 21 | | Please tell me all symptoms or illnesses that your child had in the last three months, including today. | | | | | | | | | Malaria ……………………………………….  Fever …………………………………………  Cough ……………………………………...  Difficult Breathing …………………………..  Fast Breathing/ Short Quick Breaths……...  Diarrhoea ……………………………………  Blood in stool ………………………………..  Soft or Watery stool ………………………...  Runny Nose …………………………………  Other (specify): ………… | | | | | | | | | | | | | | | | | | | | | | | | | | | | A  B  C  D  E  F  G  H  I  X | | | | | | | | | | | | | | |  | |
| 22 | | In your opinion, was your child illness mild, severe or so-so? | | | | | | | | | Mild …………………………………………..  Severe………………………………………..  so-so …………………….............................  don’t know …….…………………………….. | | | | | | | | | | | | | | | | | | | | | | | | | | | | | 1  2  3  9 | | | | | | | | | | | | | |  | |
| Check Q21:  Diarrhoea Other  28  Blood in stool  Soft or Watery stool | | | | | | | | | | | | | | | | | | | | | | | | | | | | | | | | | | | | | | | | | | | | | | | | | | | | | | | |
| 23 | | Was he/she given a fluid made from a special packet such as Jeevan Jal/Navajeevan to drink? | | | | | | | | | Yes ……………………………………………  No ………………….....................................  Don’t know ………………………………….. | | | | | | | | | | | | | | | | | | | | | | | | | | | | | 1  2  9 | | | | | | | | | | | | | | |  |
| 24 | | Was anything (else) given to treat the diarrhea? | | | | | | | | | Yes …………………….……………………..  No ……..…………......................................  Don’t know ………………………………….. | | | | | | | | | | | | | | | | | | | | | | | | | | | | | 1  2  9 | | | | | | | | | | | | | | | 26 |
| 25 | | What (else) was given to treat the diarrhea?  Anything else?  SHOW THEM PHOTOS.  RECORD ALL TREATMENTS GIVEN. | | | | | | | | | **Pill or Syrup**  Antibiotic ……..………………………………  Antimotility ………….……………………….  Zinc …………………………………………..  Other (not antibiotic, antimotility, or zinc) …  Unknown pill or syrup …….........................  **Injection**  Antibiotics …………………………………..  Non-antibiotics …………………………….  Unknown injection …………………………  (IV) Intravenous ……………………………  Home remedy/ Herbal medicine …………..  Other (specify) ……….  Don’t know ………………………………….. | | | | | | | | | | | | | | | | | | | | | | | | | | | | | A  B  C  D  E  F  G  H  I  J  K  X | | | | | | | | | | | | | | |  |
| Check Q21:  Fever/Cough Other  28  Difficult Breathing/ Runny Nose | | | | | | | | | | | | | | | | | | | | | | | | | | | | | | | | | | | | | | | | | | | | | | | | | | | | | | | |
| 26 | | At any time during the illness, did the child take any drugs for the illness? | | | | | | | | | Yes ……………………………………………  No …………………………………………….  Don’t know ……..…………………………… | | | | | | | | | | | | | | | | | | | | | | | | | | | | | 1  2  9 | | | | | | | | | | | | | | | 28 |
| 27 | | What drugs did the child take?  Any other drugs?  RECORD ALL MENTIONED.  SHOW THE PICTURE OF THESE MEDICINE. | | | | | | | | | **Antibiotic drugs**  Cotrimoxazole ……………………………..  Amoxycillin …………………………………  Ciprofloxacin ……………………………….  Procaine Penicillin injection ………………  **Other drugs**  Paracetamol ……………………………….  Ibuprofen ……………..…………………….  Cough syrup ……………………………….  Home remedy/ Harbal medicine …………  Other(specify); ……….  Don’t know …………………..…………….. | | | | | | | | | | | | | | | | | | | | | | | | | | | | | A  B  C  D  E  F  G  H  I  X | | | | | | | | | | | | | | |  |
| 28 | | Did you seek advice or treatment for your child’s illness outside the home? | | | | | | | | | Yes .............................................................  No ..............................................................  Don’t know ................................................. | | | | | | | | | | | | | | | | | | | | | | | | | | | | | 1  2  9 | | | | | | | | | | go to  32,33,34  Then 47. | | | | | |
| 29 | | Why did you decide to seek treatment outside?    DO NOT PROMPT. | | | | | | | | | Child’s health condition worsened…………  Family member advice……………………...  Advised by health facility/ doctor to do so...  Either money, transport or time became  Available ……………………………………..  Other (specify); ………….. | | | | | | | | | | | | | | | | | | | | | | | | | | | | | A  B  C  D  X | | | | | | | | | | | | | | |  |
| 30 | | How much time passed between when you first recognized that the child was ill and you took him/her to the health facility?  DO NOT PROMPT. | | | | | | | | | (days)  Don’t remember ……………………………. | | | | | | | | | | | | | | | | | | | | | | | | | | | | | 99 | | | | | | | | | | | | | | |  |
| 31 | | Where or from whom did you seek help?  If more than one provider mentioned, ask; Which provider did you go to first and which for second, after your child became ill?  DO NOT PROMPT. WITH EXPECTATION OF TRADITIONAL HEALER  (asked with sensitivity). | | | | | | | | | Mission Hospital  Health Post/  Sub health Post  PHC Outreach Clinic  FCHV ……  Pharmacy  Ayurbedic clinic  Traditional healer  Other(specify); | | | | | | | | | | | Fever  A  B  C  D  E  F  G  H  I | | | | | | | | ARI  A  B  C  D  E  F  G  H  I | | | | | | | | | | Diarrhea  A  B  C  D  E  F  G  H  I | | | | | | | | | | | | | | |  |
|  |  | FCHV not circled FCHV Circled  34 | | | | | | | | | | | | | | | | | | | | | | | | | | | | | | | | | | | | | | | | | | | | | | | | | | | | | |
| 32 | | Did you seek advice from FCHV for recent childhood illness? | | | | | | | | Yes ……………………………………………  No ……………………………………………. | | | | | | | | | | | | | | | | | | | | | | | | | | | | | | 1  2 | | | | | | | | | | | | | | | 34 |
| 33 | | If no, why did not you seek advice from FCHV for the treatment?  DO NOT PROMPT. | | | | | | | | Do not know who FCHV is …………………  FCHV was not available ……………………  Did not know FCHV provide treatment ……  FCHV is not competent …………………….  FCHV has no medicine …………………….  FCHV behavior is not good ………………..  Hospital is near ……………………………..  Other (specify): ………  Don’t know ………………………………….. | | | | | | | | | | | | | | | | | | | | | | | | | | | | | | A  B  C  D  E  F  G  H  X | | | | | | | | | | | | | | |  |
| 34 | | What are the benefits of seeking treatment from FCHV? | | | | | | | | Save time/ near the house …...……………  Can get medicine….…….…………………..  Other (specify): ………  No experience ………………………………  Don’t know ………………………………….. | | | | | | | | | | | | | | | | | | | | | | | | | | | | | | A  B  C  D  X | | | | | | | | | | | | | | |  |
| 35 | | With regard to the first (or only) provider mentioned, ask:  How satisfied were you with the service or advice provided?  Extremely satisfied? Generally satisfied? Not satisfied? | | | | | | | | Extremely Satisfied …………………………  Generally Satisfied ………………………….  Not Satisfied ………………………………… | | | | | | | | | | | | | | | | | | | | | | | | | | | | | | 1  2  3 | | | | | | | | | | | | | | |  |
| Check Q31: MORE THAN ONE PROVIDER ONLY ONE PROVIDER  38 | | | | | | | | | | | | | | | | | | | | | | | | | | | | | | | | | | | | | | | | | | | | | | | | | | | | | |  | |
| 36 | | Why did you decide to go to another provider?    DO NOT PROMPT. | | | | | | | | Child was not cured./ not satisfied with treatment ……………………………………...  Advised by health facility/ doctor to do so …  Medication was too expensive. ………..……  Instructions for treatment were too difficult to follow ……………………………………….  Other (specify): | | | | | | | | | | | | | | | | | | | | | | | | | | | | | | | | 1  2  3  4  5 | | | | | | | | | | | | |  |
| 37 | | If a second provider was mentioned, ask:  How satisfied were you with the service or advice provided by the second provider? Extremely satisfied? Generally satisfied? Not satisfied? | | | | | | | | Extremely Satisfied ……………....................  Generally Satisfied …………………………..  Not Satisfied ………………….……………… | | | | | | | | | | | | | | | | | | | | | | | | | | | | | | | | 1  2  3 | | | | | | | | | | | | |  |
| 38 | | Did you go to the health facility for your child illness? | | | | | | | | Yes ………………………………………………  No ……………………………………………….  Don’t know …………………………………….. | | | | | | | | | | | | | | | | | | | | | | | | | | | | | | | | | | | 1  2  9 | | | | | | | | | | 42 |
| 39 | | Did the health worker ask you to bring your child back to the clinic in a few days? | | | | | | | | Yes ………………………………………………  No ……………………………………………….  Don’t know …………………………………….. | | | | | | | | | | | | | | | | | | | | | | | | | | | | | | | | | | | 1  2  9 | | | | | | | | | | 42 |
| 40 | | Did you bring the child back to the health facility for a follow-up visit? | | | | | | | | Yes ………………………………………………  No ……………………………………………….  Don’t know …………………………………….. | | | | | | | | | | | | | | | | | | | | | | | | | | | | | | | | | | | 1  2  9 | | | | | | | | | | 42  42 |
| 41 | | What prevented you from taking the child for a follow-up visit?  DO NOT PROMPT. | | | | | | | | Could not afford the additional costs………………  Could not afford the time. ………………………….  Did not have the household authority to make such a decision – other household members disagreed…………………………………………….  Did not believe that follow-up was necessary ……  Has not yet taken child for follow-up, but intend …  Child became well before follow-up was required .  Other (specify); ……………….. | | | | | | | | | | | | | | | | | | | | | | | | | | | | | | | | | | | | | | | | A  B  C  D  E  F  G | | | | |  |
| 42 | | Did the health worker say your child had to be taken to a hospital or another health center? | | | | | | | | Yes ……………................................................  No …………………….…………………………..  Don’t know ………………….…………………... | | | | | | | | | | | | | | | | | | | | | | | | | | | | | | | | | | | | | 1  2  9 | | | | | | | | 47 |
| 43 | | Why did the health worker say so?  DO NOT PROMPT. | | | | | | | | Shortage of drugs …………….…………………  Shortage of staff ………………………………...  Proximity to home ………………………………  Other facility is better equipped………………..  Other (specify) ………………. | | | | | | | | | | | | | | | | | | | | | | | | | | | | | | | | | | | | | A  B  C  D  E | | | | | | | |  |
| 44 | | What did health worker recommended?  DO NOT PROMPT. | | | | | | | | Return visit to the health facility………………..  A visit to a local community health worker…….  A visit to a hospital or other health center……..  Other (specify): …………… | | | | | | | | | | | | | | | | | | | | | | | | | | | | | | | | | | | | | 1  2  3  4 | | | | | | | |  |
| 45 | | Were you able to comply? | | | | | | | | Yes ……………………………………………….  No ………………………………………………...  Don’t know ……………………………………… | | | | | | | | | | | | | | | | | | | | | | | | | | | | | | | | | | | | | 1  2  9 | | | | | | | | 47  47 |
| 46 | | If the response was “no” ask the following question.  What prevented you from taking the child for a referral?  DO NOT PROMPT. | | | | | | | | Could not afford the additional costs ……………...  Could not afford the time. ………………………….  Did not have the household authority to make such a decision – other household members disagreed. ……………………………………………  Did not believe that referral was necessary………  Has not yet taken child for referral, but intend …..  Your child became well before referral we required ………………………………………………  Other (specify): ……….. | | | | | | | | | | | | | | | | | | | | | | | | | | | | | | | | | | | | | | | | A  B  C  D  E  F  G | | | | |  |
| 47 | | When your child fell ill, and she or he was taken to a health facility, who decided that the child should seek such treatment?  DO NOT PROMPT. | | | | | | | | Respondent herself….….……..........................  Husband …………………………………………  Her father ………………………………………..  Her mother ………………………………………  Her mother-in-law ………………………………  Her father-in-law ………………………………..  Other relative ……………………………………  Community member ……………………………  Village health worker …………………………...  Doctor …………………....................................  Nurse ……………….……………………………  Other (specify) ………………. | | | | | | | | | | | | | | | | | | | | | | | | | | | | | | | | | | | | | A  B  C  D  E  F  G  H  I  J  K  X | | | | | | | |  |
| 48 | | Who took your child to the health care facility?  DO NOT PROMPT. | | | | | | | | Respondent herself….………...........................  Husband …………………………………………  Her father ………………………………………..  Her mother ………………………………………  Her mother-in-law ………………………………  Her father-in-law ………………………………..  Other relative ……………………………………  Community member ……………………………  Village health worker …………………………...  Doctor …………………….................................  Nurse …………………………………………….  Other (specify) ……………. | | | | | | | | | | | | | | | | | | | | | | | | | | | | | | | | | | | | | A  B  C  D  E  F  G  H  I  J  K  X | | | | | | | |  |
| 49 | | In general, are you able to pay the full costs involved with your children’s health care with your own income? | | | | | | | | Yes ……………………………………………….  No ………………………………………………..  Usually …………………………………………..  Sometimes ………………………………………  Don’t know …………..………………………….. | | | | | | | | | | | | | | | | | | | | | | | | | | | | | | | | | | | | | 1  2  3  4  9 | | | | | | | |  |
| 50 | | When you take a sick child to a health facility, how far must you travel to reach to health services (public hospital/ Primary health center/ sub health post/ private hospital/ pharmacy/ and Ayurbedic clinic?) Please circle distance to each facility.  Less than one hour walking? One to two hours walking? More than two hours walking? | | | | | | | | | | | | | | | | | | | | | | | | | | | | | | | | | | | | | | | | | | | | | | | | | | | | |  |
|  | |  | | Pub | PHC | | | SHP | | | | | | | | | | Private | | | | | | | Pharmacy | | | | | | | | | | | | Ayurbedic | | | | | | | | | | | | | | | | |  | |
|  |  | <1h | | 1 | 1 | | | 1 | | | | | | | | | | 1 | | | | | | | 1 | | | | | | | | | | | | 1 | | | | | | | | | | | | | | | | |  |  |
|  |  | 1-2h | | 2 | 2 | | | 2 | | | | | | | | | | 2 | | | | | | | 2 | | | | | | | | | | | | 2 | | | | | | | | | | | | | | | | |  |  |
|  |  | >2h | | 3 | 3 | | | 3 | | | | | | | | | | 3 | | | | | | | 3 | | | | | | | | | | | | 3 | | | | | | | | | | | | | | | | |  |  |
|  |  | Don’t know | | 9 | 9 | | | 9 | | | | | | | | | | 9 | | | | | | | 9 | | | | | | | | | | | | 9 | | | | | | | | | | | | | | | | |  |  |
| 51 | | Do health care workers from the health facility ever make visits in the community and/ or household? | | | | | | | | Yes ………………………………………………  No ……………………………………………….  Usually ………………………………………….  Sometimes……………………………………...  Don’t know……………………………………… | | | | | | | | | | | | | | | | | | | | | | | | | | | | | | | | | | | 1  2  3  4  9 | | | | | | | | | |  |
| 52 | | When health care instructions are communicated to you by a health worker, do you find the instructions easy to understand and follow? | | | | | | | | Yes……………………………………………….  Usually ………………………………………….  Sometimes…………….………………………..  No ……………………………………………….  Don’t know ………………............................... | | | | | | | | | | | | | | | | | | | | | | | | | | | | | | | | | | | 1  2  3  4  9 | | | | | | | | | | 54  54 |
| 53 | | If SOMETIMES or NO, ask;  What are the difficulties involved in either understanding or following such instructions?  DO NOT PROMPT. | | | | | | | | Language ……………………………………..  Literacy problems………………………………  Confusion ……………………………………..  Too much information ………………………..  Instructions not properly communicated……  Too many other concern/ pre- occupied……  Other (specify): ……… | | | | | | | | | | | | | | | | | | | | | | | | | | | | | | | | | | | A  B  C  D  E  F  G | | | | | | | | | |  |
| SECTION 4: FCHV Service Utilization | | | | | | | | | | | | | | | | | | | | | | | | | | | | | | | | | | | | | | | | | | | | | | | | | | | | | | | |
| 54 | | Do you know the FCHV who serves in your area? Do you know her name?  PROMPT: “Do you know the women who gives out Vitamin A to children under five in your area twice a year” | | | | | | | FCHV’s name:  Yes, but don’t know FCHV’s name …………..  No ……………………………………………….. | | | | | | | | | | | | | | | | | | | | | | | | | | | | | | | | | | | | | | 1  2 | | | | Finish | | | | |
| 55 | | Did your child received Vitamin A cupsule in the Vaisakh distribution from FCHV? | | | | | | | Yes ………………………………………………..  No …………………………………………………  Don’t know ………………………………………. | | | | | | | | | | | | | | | | | | | | | | | | | | | | | | | | | | | | | | 1  2  9 | | | | | | | |  |
| 56 | | Was your child given deworming tablet at that time? | | | | | | | Yes ………………………………………………...  No …………………………………………………  Don’t know ……………………………………….. | | | | | | | | | | | | | | | | | | | | | | | | | | | | | | | | | | | | | | 1  2  9 | | | | | | | |  |
| 57 | | Do you have vaccination card for your child?  If Yes, please show me.  If No, do you remember which vaccination did your child get? | | | | | | | BCG ……………...  POLIO1 ………….  POLIO2 ………….  POLIO3 ………….  DPT1 …………….  DPT2 …………….  DPT3 …………….  HEP B1 ………….  HEP B2 ………….  HEP B3 ………….  MEASLES ………. | | | | | | | | | | | | Yes  1  1  1  1  1  1  1  1  1  1  1 | | | | | | | No  2  2  2  2  2  2  2  2  2  2  2 | | | | | | | | | | Don’t know  9  9  9  9  9  9  9  9  9  9  9 | | | | | | | | | | | | | | | | |  |
| 58 | | To your knowledge, is there a Mother’s Group meeting with the FCHV in this community? | | | | | | | Yes………………………………………………..  No ………………..............................................  Don’t know………………………………………. | | | | | | | | | | | | | | | | | | | | | | | | | | | | | | | | | | | | 1  2  9 | | | | | | | | | | 61 |
| 59 | | If yes, have you ever participated in the Mother’s Group meeting? | | | | | | | Yes ……………………………………………….  No ………………………................................... | | | | | | | | | | | | | | | | | | | | | | | | | | | | | | | | | | | | 1  2 | | | | | | | | | | 61 |
| 60 | | If yes, when was the most recent Mother’s group meeting you attended? | | | | | | | Within 1 month ..………………………………..  Past 1-2 months ………………………………..  Past 2-3months …………………………………  Past 3+ months…………………………………. | | | | | | | | | | | | | | | | | | | | | | | | | | | | | | | | | | | | 1  2  3  4 | | | | | | | | | |  |
| 61 | | How long does it take you to go to FCHV’s home? | | | | | | | Minutes:  Don’t know ……………………………………… | | | | | | | | | | | | | | | | | | | | | | | | | | | | | | | | | | | | 99 | | | | | | | | | |  |
| 62 | | Please tell me which of the following kinds of help or services does your FCHV provide/ did you actually get from your FCHV/ do you expect to get from FCHV? | | | | | | | | | | | | | | | | | | | | | | | | | | | | | | | | | | | | | | | | | | | | | | | | | | | |  | |
|  |  |  | | | | | | | | | | | | | Provide | | | | | Got | | | | | | | Expect | | | | | | | | Don’t know | | | | | | | | | | | | | | | | | | |  | |
|  |  | Health information including mother’s group | | | | | | | | | | | | | 1 | | | | | 2 | | | | | | | 3 | | | | | | | | 9 | | | | | | | | | | | | | | | | | | |  |  |
|  |  | Provide advice to pregnant women | | | | | | | | | | | | | 1 | | | | | 2 | | | | | | | 3 | | | | | | | | 9 | | | | | | | | | | | | | | | | | | |  |  |
|  |  | Provide advice to post-partum mothers | | | | | | | | | | | | | 1 | | | | | 2 | | | | | | | 3 | | | | | | | | 9 | | | | | | | | | | | | | | | | | | |  |  |
|  |  | Provide advice regarding newborn | | | | | | | | | | | | | 1 | | | | | 2 | | | | | | | 3 | | | | | | | | 9 | | | | | | | | | | | | | | | | | | |  |  |
|  |  | Provide advice and treatment regarding child diarrhea | | | | | | | | | | | | | 1 | | | | | 2 | | | | | | | 3 | | | | | | | | 9 | | | | | | | | | | | | | | | | | | |  |  |
|  |  | Provide advice and treatment regarding  child respiratory infection (including pneumonia) | | | | | | | | | | | | | 1 | | | | | 2 | | | | | | | 3 | | | | | | | | 9 | | | | | | | | | | | | | | | | | | |  |  |
|  |  | Supply condom and pills | | | | | | | | | | | | | 1 | | | | | 2 | | | | | | | 3 | | | | | | | | 9 | | | | | | | | | | | | | | | | | | |  |  |
|  |  | Distribute Iron tablet for pregnant women | | | | | | | | | | | | | 1 | | | | | 2 | | | | | | | 3 | | | | | | | | 9 | | | | | | | | | | | | | | | | | | |  |  |
|  |  | Distribute Vitamin A for post-natal | | | | | | | | | | | | | 1 | | | | | 2 | | | | | | | 3 | | | | | | | | 9 | | | | | | | | | | | | | | | | | | |  |  |
|  |  | Provide HIV/AIDS/STI information | | | | | | | | | | | | | 1 | | | | | 2 | | | | | | | 3 | | | | | | | | 9 | | | | | | | | | | | | | | | | | | |  |  |
| 63 | | When was the last time you got services from FCHV? | | | | | | | | | | Weeks ago  Don’t remember …………………………….  Never met ……………...…………………… | | | | | | | | | | | | | | | | | | | | | | | | | | | | | | | | | 98  99 | | | | | | Finish | | | | |
| 64 | | How do you satisfy with FCHV service?  Extremely satisfied? Generally satisfied? Not satisfied? | | | | | | | | | | Extremely satisfied ……………………………  Generally satisfied …………………………….  Not satisfied ……………………………………  Don’t know/ never get service ………………. | | | | | | | | | | | | | | | | | | | | | | | | | | | | | | | | | 1  2  3  9 | | | | | | | | |  | |
| 65 | | Please tell me which of the following part of services should be improved for you to use FCHV services for child illness treatment more frequently? | | | | | | | | | | Increase access to FCHV…………………….  Improve service quality ……………………….  Medicine availability …………………………..  Improve FCHV’s interpersonal mannar……...  Other (specify): ….  Don’t know ………………..…………………… | | | | | | | | | | | | | | | | | | | | | | | | | | | | | | | | | A  B  C  D  E  X | | | | | | | | |  | |
